# Hiccups triggered by bladder filling after bilateral pontine hemorrhage: A case report

**DOI:** 10.1097/MD.0000000000019338

**Published:** 2020-04-03

**Authors:** Jinmann Chon, Seung Don Yoo, Seung Ah Lee

**Affiliations:** Department of Physical Medicine and Rehabilitation, Kyung Hee University College of Medicine, Seoul, Republic of Korea.

**Keywords:** hiccup, neurogenic, stroke, urinary bladder

## Abstract

**Introduction:**

A hiccup is myoclonus of a sudden involuntary contraction of the diaphragm. Hiccups have various causes, and brain stem stroke is one of the causes of central hiccups. Certain types of hiccups are caused by diseases that can be fatal. Therefore, it is beneficial for physicians to be familiar with the various cases of unusual hiccups. We report a case of hiccups triggered by urinary bladder filling in a brain stem stroke patient. To the best of our knowledge, previous reports have not described a similar case.

**Patient concerns:**

We describe the case of a 54-year-old patient who had acute bilateral pontine hemorrhage. The patient had intermittent hiccups in the early stages of the stroke onset. The hiccups ceased by the administration of medication or stimulation of the pharyngeal or tracheal wall. Two months after the onset, the Foley catheter was removed to check if the patient could void the bladder voluntarily. Hiccups occurred whenever the bladder was filled with some amount of urine.

**Diagnosis:**

Pontine hemorrhage, neurogenic bladder, and quadriplegia.

**Interventions:**

When the hiccups occurred, the amount of urine in the bladder was checked using a transabdominal bladder ultrasonography scanner. After clean intermittent catheterization for bladder emptying, the hiccups subsided.

**Outcomes:**

The hiccups occurred 5 or 6 times a day, as often as the bladder was filling. He was unable to void the urine voluntarily for 5 days after the removal of the Foley catheter. Percutaneous suprapubic cystostomy was performed finally to remove the stimulation of bladder filling and the hiccups disappeared.

**Conclusion:**

Bladder filling is suspected to increase the sympathetic tone and cause a hiccup reflex. Bladder filling could be a factor triggering hiccups in pontine hemorrhage.

## 1 Introduction

A hiccup is abrupt erratic diaphragmatic muscular contraction, and is followed by laryngeal closure immediately.^[1]^

Hiccup is usually a self-limited disorder; hence, many episodes of hiccups may subside spontaneously without any clinical significance. Most hiccups are brief self-limiting episodes of no clinical significance. The most frequent benign causes are gastric distension (e.g., overeating, eating too fast, drinking carbonated drinks, and aerophagia), a sudden change in temperature (e.g., very hot or cold food or drinks, and a cold shower), drinking alcohol, excessive smoking, or psychogenic (sudden excitement or emotional stress).^[2]^ To date, no report has discussed that bladder filling causes hiccups.

The pathophysiology of hiccupping is poorly understood, but it may be mediated through a hiccup reflex arc. A reflex arc is involved with phrenic nerve, vagus nerve, and sympathetic pathways and central modulation are likely responsible for hiccups. Accordingly, any irritant, such as physical/chemical factors, inflammation, and neoplasia invading the arc leads to hiccups. The central causes of hiccups include stroke, space-occupying lesions, injury, and others. Among the types of stroke, brain stem stroke is the most common cause of hiccups. We present the case of a middle-aged man who had hiccups repeatedly every time his bladder was filling, after bilateral pontine hemorrhage. We performed a literature review of the previous cases and aimed to elucidate a possible mechanism for our case. The patient's guardian provided informed consent for participation in the study.

## 2 Case report

**Figure 1:**
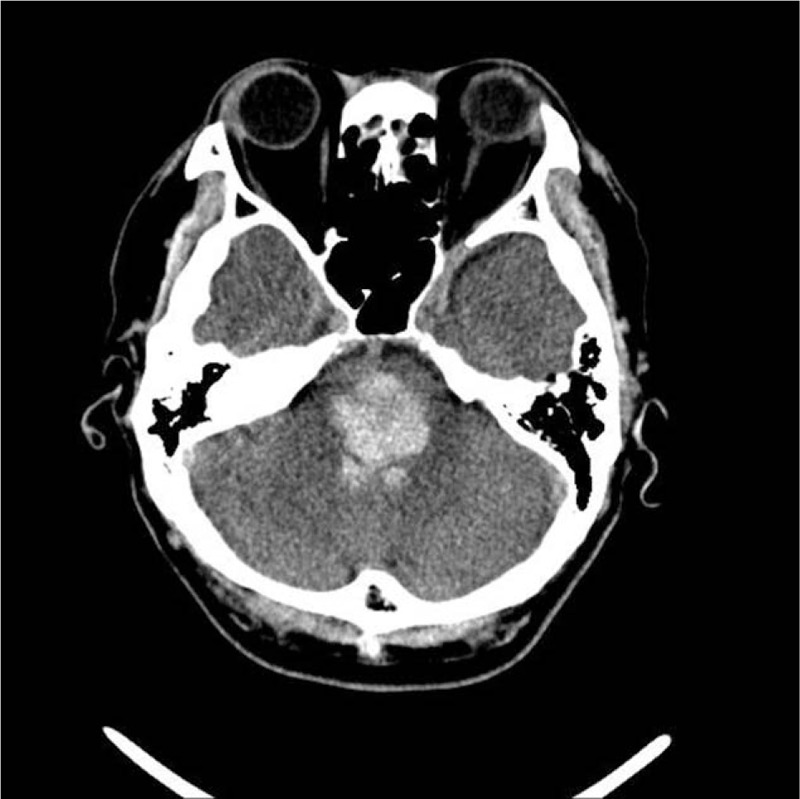
Computed tomography (CT) scan shows high attenuation in the acute pontine hemorrhage with intraventricular hemorrhage at the fourth ventricle.

A 54-year-old man without any premedical history had sudden-onset left-sided weakness at home. A few minutes later, right-sided weakness also developed, and he could not speak. He was admitted to the emergency department in a stuporous state. The initial Glasgow coma scale score was 4 (extensor posturing to pain, no eye opening, and no verbal response). The initial brain computed tomography scan revealed acute pontine hemorrhage with intraventricular hemorrhage at the fourth ventricle (Fig. 1).

In the intensive care unit, he remained in a semicoma, and central high fever and apnea developed, which could occur in pontine hemorrhage. A Foley catheter, Levin tube, and endotracheal tube were inserted. After 7 days in the hospital, he opened his eyes spontaneously but was not aware of his surroundings. Making eye contact was impossible, and he could not move his extremities at all. Hiccups occurred once every few days. In addition, the hiccups subsided after administration of metoclopramide HCl or stimulation of the pharyngeal and tracheal walls with a suction catheter.

On the 70th day of hospitalization, the Foley catheter was removed to check if he could urinate voluntarily. Six hours after the removal, hiccups began again. Stimulation of the pharyngeal and tracheal walls was not successful to cease the hiccups. At that time, urine volume was assessed using a transabdominal ultrasonography bladder scan. The urine volume was 260 mL. Two hours later, although the urine volume increased up to 360 mL, he could not void voluntarily. Bladder emptying was performed through clean intermittent catheterization (CIC). Within a few minutes after the CIC, the hiccups that lasted for 2 hours spontaneously subsided. Ever since, whenever his bladder was filled with urine at a volume of as much as 175 to 350 mL, hiccups occurred. After the CIC, the hiccups were terminated. He could not void urine voluntarily for 5 days after the removal of the Foley catheter. Thus, the physician decided to reinsert a Foley catheter for continuous drainage of urine. Hiccups did not occur thereafter. In consultation with a urologist, percutaneous suprapubic cystostomy was the final decision to remove the stimulation of bladder filling. Three months after a stroke, suprapubic cystostomy was performed. He discharged from hospital 5 days after suprapubic cystostomy procedure. One and a half years after a stroke, motor power did not recover and urinary drainage maintained through the suprapubic cystostomy without hiccups.

## 3 Discussion

We report a case of a hiccup induced by bladder filling after pontine hemorrhage. Approximately 4000 hospital admissions due to hiccups are reported every year in the United States.^[1]^ Hiccups are more common in central nervous system and gastrointestinal diseases. Persistent or intractable hiccup is a common disorder that can occur due to brain stem lesions. Keane's^[3]^ analysis revealed that 56% of his experience with central hiccups were secondary to lateral medullary infarcts. However, any ischemic insult to the brainstem or pons^[4]^ will place an individual at risk of developing intractable hiccups. If hiccups persist, aspiration, during episodes of regurgitation secondary to hiccups, and loss of airway control would occur. It is more fatal to stroke patients. Therefore, prompt and appropriate management is required.

The production of hiccups is a complex mechanism that involves multiple neurotransmitters and anatomical structures within the central and peripheral nervous systems. The reflex arc has afferents from the phrenic, vagus, and sympathetic nerves (T6–T12) to the central processing area in the brain stem, with efferents in the motor fibers of the phrenic nerve to the diaphragm and accessory nerve to the intercostal musculature.^[2,5,6]^ Brain stem infarction may cause disintegration of inhibitory connections with a higher central pathway, which could make hiccups persist.

Considering the bladder neurophysiology, connections between various brain areas and extensive tracts in the spinal cord including sympathetic, parasympathetic, and somatic systems are necessary for the neural control of micturition. In the brain, the pontine micturition center (PMC, Barrington nucleus) is specific for the micturition center.^[7]^ In an intact central circuit, excitation of the PMC activates the descending pathways that control urethral sphincter relaxation and activation of the sacral parasympathetic outflow. This results in bladder contraction and increased intravesical pressure and urinary flow.

During the bladder filling phase, the sympathetic afferent fibers are activated with somatic afferent fibers owing to the mechanical distension of the bladder wall. In this case, we postulated that the hiccups were caused by the irritative sympathetic fibers of the hiccup reflex arc that were full-blown at the end of the filling phase. If the PMC was intact and the bladder emptying phase was initiated, the parasympathetic tone would be activated, and the sympathetic tone would be turned off. As the PMC was injured due to the stroke, the sympathetic tone subsided, and in turn, the hiccups subsided after artificial bladder emptying.

If hiccups in pontine stroke patients are persistent, physicians must check for intra-abdominal stimuli, especially bladder distension.

## Author contributions

**Conceptualization:** Jinmann Chon, Seung Ah Lee, Seung Don Yoo.

**Data curation:** Jinmann Chon, Seung Ah Lee.

**Investigation:** Seung Ah Lee.

**Methodology:** Seung Ah Lee.

**Supervision:** Seung Ah Lee.

**Writing – original draft:** Jinmann Chon, Seung Ah Lee, Seung Don Yoo.

**Writing – review & editing:** Jinmann Chon, Seung Ah Lee, Seung Don Yoo.

Seung Ah Lee orcid: 0000-0002-3426-6259.

## References

[1] Cymet TC Retrospective analysis of hiccups in patients at a community hospital from 1995–2000. J Natl Med Assoc 2002;94:480–3. 12078929 PMC2594386

[2] Wilcox SK Garry A Johnson MJ Novel use of amantadine: to treat hiccups. J Pain Symptom Manage 2009;38:460–5. 19735905 10.1016/j.jpainsymman.2008.10.008

[3] Keane JR Hiccups due to central nervous system disease: analysis of 71 inpatients. Can J Neurol Sci 2010;37:870–2. 21059556 10.1017/s0317167100051611

[4] Kumar A Dromerick AW Intractable hiccups during stroke rehabilitation. Arch Phys Med Rehabil 1998;79:697–9. 9630152 10.1016/s0003-9993(98)90047-8

[5] Nausheen F Mohsin H Lakhan SE Neurotransmitters in hiccups. Springerplus 2016;5:1357. 27588250 10.1186/s40064-016-3034-3 PMC4988959

[6] Steger M Schneemann M Fox M Systemic review: the pathogenesis and pharmacological treatment of hiccups. Aliment Pharmacol Ther 2015;42:1037–50. 26307025 10.1111/apt.13374

[7] Holstege G Micturition and the soul. J Comp Neurol 2005;493:15–20. 16254993 10.1002/cne.20785

